# Composite Materials Based on Iron Oxide Nanoparticles and Polyurethane for Improving the Quality of MRI

**DOI:** 10.3390/polym13244316

**Published:** 2021-12-09

**Authors:** Luiza Madalina Gradinaru, Mihaela Barbalata Mandru, Mioara Drobota, Magdalena Aflori, Maria Butnaru, Maria Spiridon, Florica Doroftei, Mihaela Aradoaei, Romeo Cristian Ciobanu, Stelian Vlad

**Affiliations:** 1 “Petru Poni” Institute of Macromolecular Chemistry, Gr. Ghica Voda Alley, 41A, 700487 Iasi, Romania; mihaelamandru84@gmail.com (M.B.M.); miamiara@icmpp.ro (M.D.); maflori@icmpp.ro (M.A.); mariabutnaru@yahoo.com (M.B.); dana_spiridon_is@yahoo.com (M.S.); florica.doroftei@icmpp.ro (F.D.); 2 Department of Biomedical Sciences, “Grigore T. Popa” University of Medicine and Pharmacy, Kogalniceanu Street, 9-13, 700115 Iasi, Romania; 3 Electrical Engineering Faculty, “Gheorghe Asachi” Technical University of Iasi, Dimitrie Mangeron Bd., 67, 700050 Iasi, Romania; mihaela.aradoaei@academic.tuiasi.ro (M.A.); rciobanu@yahoo.com (R.C.C.); 4 SC All Green SRL, I. Bacalu Street, 5, 700029 Iasi, Romania

**Keywords:** polyurethane, iron oxide nanoparticles, composites, MRI

## Abstract

Polyether urethane (PU)-based magnetic composite materials, containing different types and concentrations of iron oxide nanostructures (Fe_2_O_3_ and Fe_3_O_4_), were prepared and investigated as a novel composite platform that could be explored in different applications, especially for the improvement of the image quality of MRI investigations. Firstly, the PU structure was synthetized by means of a polyaddition reaction and then hematite (Fe_2_O_3_) and magnetite (Fe_3_O_4_) nanoparticles were added to the PU matrices to prepare magnetic nanocomposites. The type and amount of iron oxide nanoparticles influenced its structural, morphological, mechanical, dielectric, and magnetic properties. Thus, the morphology and wettability of the PU nanocomposites surfaces presented different behaviours depending on the amount of the iron oxide nanoparticles embedded in the matrices. Mechanical, dielectric, and magnetic properties were enhanced in the composites’ samples when compared with pristine PU matrix. In addition, the investigation of in vitro cytocompatibility of prepared PU nanocomposites showed that these samples are good candidates for biomedical applications, with cell viability levels in the range of 80–90%. Considering all the investigations, we can conclude that the addition of magnetic particles introduced additional properties to the composite, which could significantly expand the functionality of the materials developed in this work.

## 1. Introduction

In recent years, the molecular imaging technique has attracted much interest due to its non-invasive procedure, which consists of a combination of in vivo imaging and molecular biology, aiming at the identification of living biological process at the cellular and molecular level [1]. Thus, molecular imaging permits quantitative characterization that allows the non-invasive monitoring and early diagnosis of diseases, and is mainly used for cell tracking, angiogenesis, apoptosis, and in vivo tissue gene imaging [2].

Magnetic resonance imaging (MRI) is one of the major imaging methods because of the combination of several advantages: convenient non-invasive application, high spatial resolution, and tomographic capability. MRI shows its great supremacy in the practical application of clinical diagnosis, as well as in biomedical research [3], and is mainly used to obtain high quality images of the inside of the human body. MRI has the advantages of relatively high resolution (25 ÷ 100 μm) and superior tissue penetration depth, while its sensitivity requires substantial improvement compared with the direct optical imaging technique [4]. Moreover, this technique is based on the principles of nuclear magnetic resonance (NMR), a spectroscopic technique used by researchers to obtain chemical and physical information about molecules [2]. However, as with any other imaging modality, MRI is vulnerable to artifacts that arise for different reasons, such as data acquisition, image reconstruction processes, scanning parameters, etc. Various correction methods have been developed to mitigate the corruptive effects of artifacts and to improve image diagnostic quality, most of them regarding the procedure, equipment, or postprocessing algorithms [5]. Another drawback of this method consists in the use of contrast agents that could provoke a series of hemodynamic changes, anaphylactic reactions, or side effects due to their oral or intravascular administration [3,4,6].

Since inorganic nanoparticles commonly have remarkably high thermal and mechanical properties, a wide range of them have been used as fillers in different types of polymer matrices to improve their properties [6,7,8,9]. Their nanoscale dimensions lead to high surface energy, which allows efficient mixing with a polymer matrix at the molecular level. Among inorganic nanoparticles, magnetic nanoparticles have unique multifunctional properties and numerous applications [6,10].

Iron oxide nanoparticles, which belong to the ferromagnetic class of magnetic materials, have attracted incredible interest since they are biocompatible and show exceptional electric, thermal, mechanical, and magnetic properties and, thus, are utilized for many different biomedical and bioengineering applications [6,11,12]. The most common iron oxide forms available are hematite (α-Fe_2_O_3_), maghemite (γ-Fe_2_O_3_) and magnetite (Fe_3_O_4_), which are used in different technological, industrial, and biomedical applications [13].

Incorporating different types of iron oxide nanoparticles into organic polymers to afford nanocomposites is of great interest because of the versatile properties of this class of organic–inorganic composite. Moreover, the preparation of composites with magnetic properties allows the production of some functional complex components such as electromagnetic screening devices and other electrical systems [14].

Among the different polymer matrices that can be used to embed nanoparticles, polyurethanes (PU) are of particular interest because of their wide range of applications in different fields, including as textiles, adhesives, and coatings in the construction, automotive, and especially biomedical engineering fields [15,16,17,18,19]. PUs are multi-segmented polymers, prepared from the reaction of polyols, diisocyanates, and diols. Therefore, PUs have alternating hard and soft blocks that lead to microphase separation under appropriate conditions, possessing elastomeric character and extra strength. This polymer type is capable of easily changing its properties by varying the chemical composition or adding filler reinforcement agents [20]. A significant effort has been made to develop new strategies to obtain different magnetic PU materials that can be used as adhesives [21], as encapsulating materials for flexible organic photovoltaic cells [22], as coatings [23], in decontamination/filtration [24,25,26], in the preparation of microwave-absorbing materials [27], and especially in biomedical applications [20,28,29]. The incorporation of nanoparticles into the PU matrix has the following advantages: a structural support and a functionalization of the matrix is provided by increasing the dispersibility of the nanoparticles and, secondly, their incorporation confers magnetic properties and antibacterial activity to the polymer [12]. Moreover, the presence of iron oxide nanoparticles into polyurethane matrix can change the molecular structures through interaction with polar groups of the polyurethane via different inter- and intramolecular interactions. They act as a cross-linking agent leading to ordered and reinforced nanocomposite structures [20,30]. Studies confirmed that PU embedded with iron oxide nanoparticles were utilized to improve some characteristics of PUs, such as electric [15], magnetic [10], thermal [13], mechanical [31,32,33], and shape memory [31] properties. Properties such as crystallinity, mechanical strength, surface roughness, hydrophilicity, and eventually cell fates can be changed through the incorporation of such nanoparticles to the PU matrix [20]. Moreover, by combining the properties of the polyurethane structures such as high elasticity, good conductibility, stability, and biocompatibility, with those of iron oxide nanoparticles, especially magnetization, novel composites could be developed to improve the image quality in MRI investigations.

The aim of the present study was to synthesize and characterize novel magnetic nanocomposites based on the use of poly(ether-urethane) as the matrix and two types of iron oxide nanoparticles (Fe_2_O_3_ and Fe_3_O_4_) as the nano-sized fillers. This is the first step in the development of such materials that could be explored as custom-tailored composites for the preparation of suitable devices to improve the image quality in MRI investigations, when, for various reasons, defects in the clarity of images appear. This device should act as an image amplifier that should be placed at the points where the MRI image does not cover areas of vital interest—for example, in the case of brain tumours. Therefore, pristine and nanocomposite PU films were prepared via solution casting by embedding different weight percentages (0.1; 0.5; 1%) of each Fe_2_O_3_ and Fe_3_O_4_ nanoparticle into PU matrices (30 wt.% in DMF) through blending. Then, the influence of the nanoparticles’ amounts on the structural, morphological, mechanical, dielectric, and magnetic properties was investigated. In addition, in vitro cytocompatibility was also verified by MTT assay and cell.morphology.

## 2. Materials and Methods

### 2.1. Materials

Poly(tetrahydrofuran) (Terathane polyether glycol) (PTHF) with M_n_ 2000 g/mol, 1,4-butanediol (BD), poly(dimethylsiloxane) bis(hydroxyalkyl) terminated (PDMS) with M_n_ 5600 g/mol and N,N-dimethylformamide (DMF) were purchased from Sigma-Aldrich (Steinheim, Germany). 4,4′-diphenylmethane diisocyanate (MDI) was obtained from Fluka (Steinheim, Germany) and was fresh distilled prior to use. Iron (III) oxide nano powder (Fe_2_O_3_) of <50 nm particle size and Iron (II, III) oxide nano powder (Fe_3_O_4_) of 50–100 nm particle size (SEM) were used as the magnetic particles throughout the investigation and were also purchased from Sigma-Aldrich (Steinheim, Germany).

For determination of contact angle values, four test liquids with different surface tensions [34,35] were selected: Milli-Q purified water (Millipore, ≥18.2 MΩ cm), ethylene glycol (anhydrous 99.8%, Sigma-Aldrich, Steinheim, Germany), formamide (99%, Sigma-Aldrich, Steinheim, Germany), and methylene iodide (99%, Sigma-Aldrich, Steinheim, Germany). All other chemicals were used as received without further purification.

### 2.2. Preparation Methods 

#### 2.2.1. Synthesis of Pristine PU

**Scheme 1:**
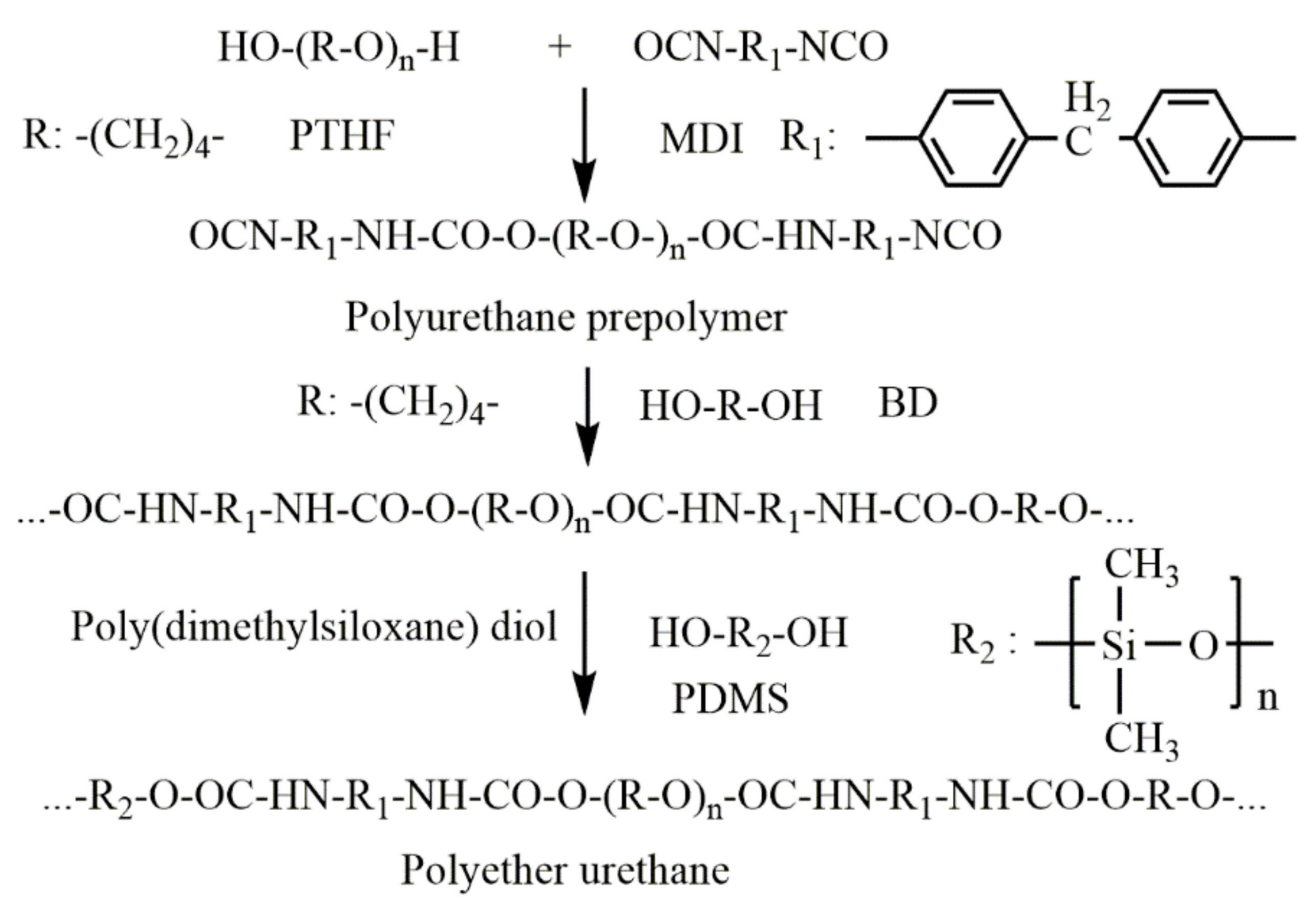
The schematic illustration of the synthesis route of pristine PU.

PU was synthetized using a polymerization solution method based on a previously reported procedure [36,37]. Briefly, in a four-necked glass reaction vessel equipped with a stirrer, a dropping funnel, and an N_2_ inlet and outlet, one equivalent quantity of PTHF was dehydrated under vacuum (1–3 mmHg) and temperature (100 °C) for 4 h. Then, the reaction vessel was brought to atmospheric pressure under nitrogen and the temperature was decreased until 80 °C was reached. Then, two equivalent quantities of MDI were added and stirred at 80 °C for 2 h. After two dilution steps, the prepolymer was extended with the required amount of BD and PDMS solution in DMF. The molar ratio of the reactants PTHF:MDI:BD/PDMS was 1:3:2 (Scheme 1).

#### 2.2.2. Preparation of Fe_2_O_3_ and Fe_3_O_4_ PU Nanocomposites

Six different Fe_2_O_3_ and Fe_3_O_4_ PU nanocomposites were prepared by embedding different weight percentages (0.1; 0.5; 1%) of each Fe_2_O_3_ and Fe_3_O_4_ nanoparticle into PU matrices (30 wt.% in DMF) through blending. Thereby, the dispersions were stirred vigorously for 2 h and then were ultrasonicated for 6 h to obtain stable and homogeneous suspensions. Pristine and nanocomposite PU films (approximately 0.2 mm in thickness) were prepared by solvent casting of the final suspension on a glass plate followed by drying at room temperature and low pressure (1–2 mmHg) for 24 h. Sample code designations and formulations of pure PU and nanocomposites are given in Table 1.

### 2.3. Characterization Technique

#### 2.3.1. Fourier Transform Infrared–Attenuated Total Reflectance (FTIR-ATR) Spectroscopy

The infrared spectra of pristine and PU nanocomposites were recorded using a Bruker LUMOS-FT-IR Microscope (Bruker Optik GmbH, Ettlingen, Germany) with an ATR reflection module (Attenuated Total Reflection) and a diamond crystal, at a single reflection angle of 45° equipped with OPUS 8 software for spectral processing. The surfaces were scanned in the 500–4000 cm^−1^ range with a resolution of 2 cm^−1^, with averaging over 64 scans. The spectra were recorded at a constant temperature of 25 °C.

#### 2.3.2. Mechanical Analysis

Tensile tests of the dumbbell-shaped pristine and nanocomposite PU strips (50 mm × 8.5 mm × 4 mm) were carried out at room temperature (20–22 °C), using a universal testing machine (Instron, Norwood, MA, USA), operated with a speed of 30 mm/min. Three replicates were used for each sample to obtain the averaged values and standard deviation. Elongation at break and tensile strength values were directly determined from the stress–strain curves. The Young’s modulus was evaluated from the slope of the stress–strain curve in the linear region at low deformation.

#### 2.3.3. Scanning Electron Microscopy (SEM) Analysis

The surface morphology, dispersion of the nanoparticles in PU nanocomposites, and elemental composition were evaluated with a Verios G4 UC scanning electron microscope (Thermo Scientific, Waltham, MA, USA) equipped with an energy-dispersive x-ray spectroscopy analyser (Octane Elect Super SDD detector(AMETEK, Tokyo, Japan). Before image acquisition, the samples were coated with 10 nm platinum using a Leica EM ACE 200 Sputter Coater (Leica Microsystems, Vienna, Austria) to provide electrical conductivity and to prevent charge build up during exposure to the electron beam. SEM investigations were performed in High Vacuum mode using a secondary electron detector (Everhart–Thornley detector, ETD) at an accelerating voltage of 5 kV. The pore diameters were determined from SEM images using ImageJ software. From each image, at least 50 different pores were randomly selected, and their diameters were measured to generate an average.

#### 2.3.4. Surface Topography

A stylus profiler Tencor Alpha-Step D-500 was used for studying the surface topography of the samples with the amplitude parameters (KLA Tencor Corporation, Milpitas, CA, USA), such as the average roughness (Ra) and the root mean square roughness (Rq). The measured heights of the steps had an accuracy ranging between 10 Å and 1.2 mm. The device measured the roughness parameters with a recording speed of 0.10 mm/s and a filtration interval of 0.060 mm. The average roughness (Ra) was calculated as representative roughness information. The reported Ra values were the average of 5 readings corresponding to different places and different directions.

#### 2.3.5. Contact Angle Determination and Surface Wettability Study

Static contact angle (SCA) measurements were also implemented via the sessile drop method using a CAM 101 Optical Contact Angle Instrument (KSV Instruments, Helsinki, Finland) equipped with a special optical system (charge-couple device—CCD) connected to a computer. SCA measurements of distilled and deionized water (W), ethylene glycol (EG), formamide (FA), and di-iodomethane (dIM) were measured to examine the wettability of the PU surface samples with or without iron oxide nanoparticles. Five measurements conducted on different parts of the samples were averaged. Measurements were performed on dried surfaces at room temperature. 

Surface tension parameters of the samples were estimated adopting the method developed by Owens, Wendt, Rabel, and Kaelble [35], based on the SCA values, as shown in the following equation:

(1) $$\frac{1+\cos\theta }{2}\;\cdot \;\frac{\gamma_{lv}}{\sqrt{\gamma_{lv}^{d}}}=\sqrt{\gamma_{sv}^{p}}\;\cdot \;\sqrt{\frac{\gamma_{lv}^{p}}{\gamma_{lv}^{d}}}+\sqrt{\gamma_{sv}^{d}}\;;\;\gamma_{sv}=\gamma_{sv}^{d}+\gamma_{sv}^{p}$$

where *θ* is the SCA determined with the test liquids, the subscripts “*lv*” and “*sv*” denote the interfacial tension between liquid-vapour and surface-vapour, respectively, while the superscripts “*p*” and “*d*” denote the polar and disperse components, respectively, of the total surface tension *γ_sv_*.

The interfacial solid−liquid tension (*γ_sl_*) is calculated based on the following equation:

(2) $$\gamma_{sl}=\gamma_{sv}+\gamma_{lv}-2\left(\sqrt{\gamma_{sv}^{p}\gamma_{lv}^{p}}+\sqrt{\gamma_{sv}^{d}\gamma_{lv}^{d}}\right)$$

The critical surface tension (*γ_c_*) was estimated using Zisman’s method [35] and it was derived from a plot of cos *θ* versus the surface tension of various test liquids. The linear regression extrapolated value of this tension at cos *θ* = 1 gave *γ_c_* and represented the maximum surface tension of a liquid that could completely wet the substrate.

The work of adhesion (*W_a_*) was calculated using Young–Dupre equation [35,38]:

(3) $$W_{a}=\gamma_{lv}\left(1+\cos\theta \right)$$

The spreading coefficient (*S*) can also be calculated as the difference between the adhesion (*W_a_*) and cohesion (*W_c_*) work [39].

(4) $$S=W_{a}-W_{c}$$

where $W_{c}=2\gamma_{lv}$.

#### 2.3.6. Determination of Dynamic Vapours’ Sorption and Diffusion Coefficients

The dynamic vapours’ sorption (DVS) capacity of the nanocomposite samples was measured in a dynamic regime using an automated gravimetric analyser IGAsorp apparatus (Hiden Analytical, Warrington, UK). The IGAsorp is a standard sorption apparatus, equipped with a sensitive microbalance (resolution 1mg and capacity 200 mg), which continuously registers the weight of the sample together with the temperature and relative humidity. Based on the experimental isotherm data of the sorption–desorption experiment at 25 °C, the diffusion coefficients could be determined using Fick’s first and second laws. Crank [40] deduced that the diffusion coefficient for short time periods (M_t_/M_∞_ < 0.5) is described by Equation (5):

(5) $$\frac{M_{t}}{M_{\infty }}=\frac{4}{l}\sqrt{\frac{D_{1}\cdot t}{\pi }}$$

where *M_t_* (g) is the mass of sorbed water vapour at time *t* (s), *M_∞_* (g) is the mass sorbed at *t* = ∞, l (cm) is the sample thickness and *D* (cm^2^/s) is the Fickian diffusion coefficient. The Fickian diffusion coefficient of the water vapour was determined from the initial slope of the (*M_t_/M_∞_*)^2^ versus *t*^1/2^ plot (extracted from kinetic sorption experiments). At longer time (*M_t_/M_∞_* > 0.5), the Fickian diffusion coefficient could be deduced from Equation (6):

(6) $$\frac{M_{t}}{M_{\infty }}=1-\frac{8}{\pi^{2}}e^{\frac{-D_{2}\pi^{2}t}{l^{2}}}$$.

#### 2.3.7. Dielectric Analysis

The equipment for determining the dielectric measurements consisted of the following: anAlpha-N Frequency Analyzer from Novocontrol GmBH, Montabaur, Germany; a Rhode–Schwartz NVR Network Analyzer (Milpitas, CA, USA), which worked in the frequency range of 20 kHz ÷ 8 GHz, impedance 0.1 Ω … 10 kΩ, tan (δ) accuracy > 3×10^−2^; a QUATRO-Power temperature control unit (Novocontrol GmBH, Montabaur, Germany), with an accuracy of 0.01 °C and stabilization with an accuracy of 0.1 °C; and the WinDETA/WinFIT software packages for measurement, calibration, and analysis. The dielectric characteristics as a function of frequency were studied at the temperature of 25 °C.

#### 2.3.8. Magnetic Determination

The assessment of the magnetic properties of the samples was performed at ambient temperature, using an MPMS3 (7 T) SQUID vibrating-sample magnetometer (VSM) operated in DC mode (Lake Shore Cryotronics, Woburn, MA, USA. The magnetic field was swept from −30,000 to 30,000 Oe with a stepwise progression of 50 Oe.

### 2.4. In Vitro Evaluation of Biocompatibility—MTT Assay

Initial preliminary cytotoxicity tests were performed using a mammalian MCF 7 epithelial cell line (purchased from European Collection of Cell Cultures (ECACC)) at a concentration of 20 × 10^4^ cells/well and a yellow 3-(4,5-dimethylthiazol-2-yl)-2,5-diphenyl tetrazolium bromide (MTT) assay. This is a quantitative colorimetric method, based on the capacity of the cell to reduce the yellow tetrazolium salts to intense purple formazan by means of an intracellular reduction system mostly located in the mitochondria. The MTT assay is widely used for the assessment of cell viability and proliferation studies [41]. Briefly, after incubation of PU materials with cells for 48 and 72 h, the MTT solution (0.25 mg/mL) was added and incubated with for 3 h in the dark, at 37 °C. After incubation, formazan crystals were solubilized with isopropanol and quantified by spectrophotometry at 570 nm. The amount of formazan was correlated with number of viable cells. The cell viability was normalized to that of epithelial cells cultured in the media with negative control (without material). The experiments were performed in triplicate and the results are presented as mean ± standard deviation (SD).

The morphology of the cells was also examined, after 72 h of direct contact of the samples with cells, by fluorescence and phase-contrast microscopy. The cells were fixed with an aqueous solution of formaldehyde and stained with 2-(4-amidinophenyl)-1H -indole-6-carboxamidine (DAPI) for nuclei observation. The blue fluorescence of the cell nuclei was detected using a 358/461 nm excitation/emission filter with a Leica DMIL inverted microscope (Leica, Wetzlar, Germany).

### 2.5. Statistical Analysis

For tests that produced numerical values, the test results were expressed as mean ± standard deviation (SD). For results obtained from different groups or different time points, statistical analysis was performed using one-way analysis of variance (ANOVA). The *p* value < 0.05 was considered to be statistically significant.

## 3. Results and Discussion

### 3.1. FTIR-ATR Analysis

**Figure 1:**
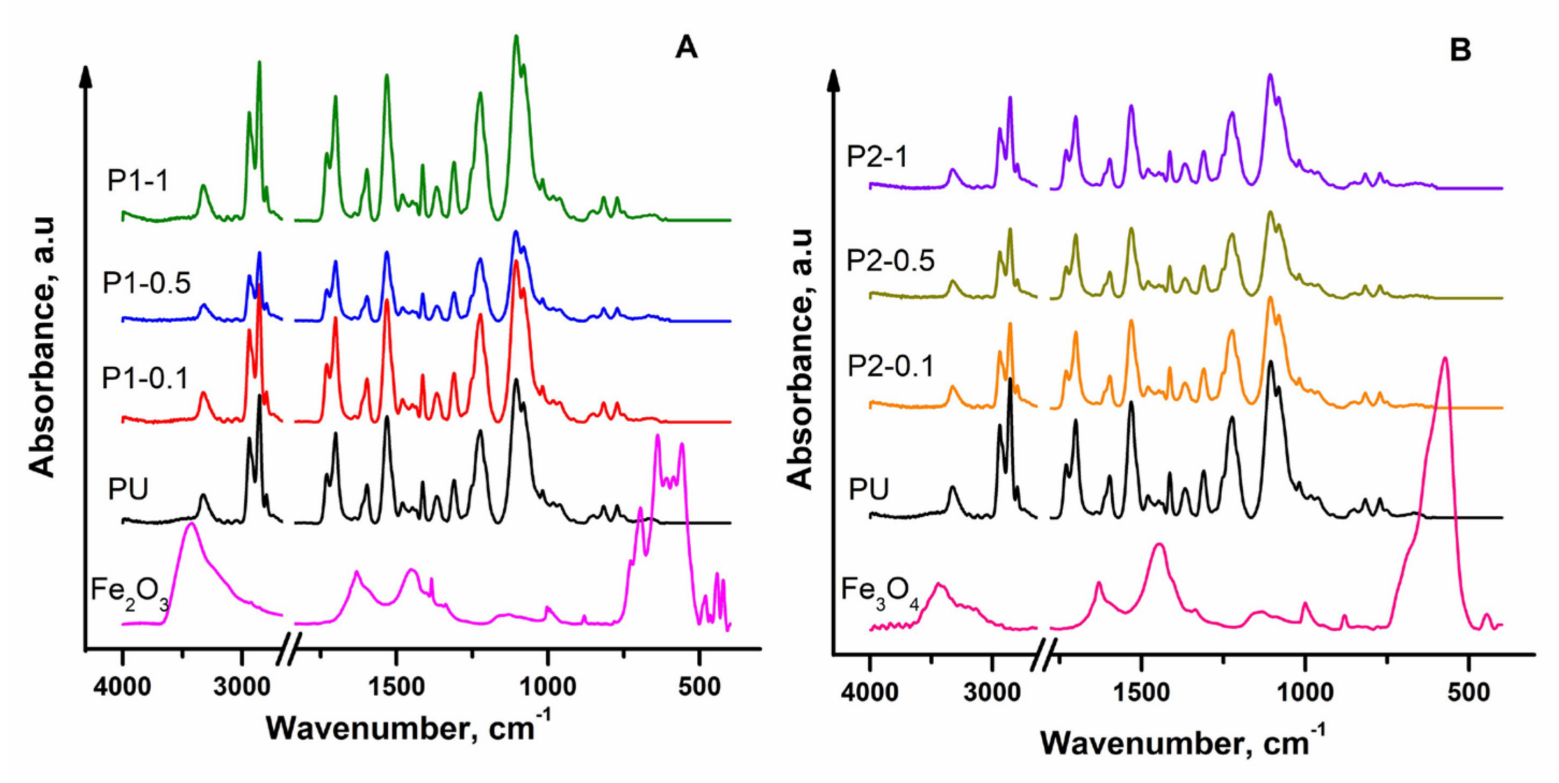
Comparison of ATR-FTIR spectra of: (**A**) pure Fe_2_O_3_ nanoparticles, pristine PU, and PU nanocomposites embedded with different amounts of Fe_2_O_3_ nanoparticles, and (**B**) pure Fe_3_O_4_ nanoparticles, pristine PU, and PU nanocomposites embedded with different amounts of Fe_3_O_4_ nanoparticles.

IR spectroscopy is an important and popular investigation technique that provides very useful information about the chemical composition and molecular structure, and also about the structure–properties relationship. The pure and PU nanocomposite samples were examined by FTIR-ATR analysis and, in Figure 1, the IR spectra of pure Fe_2_O_3_ and Fe_3_O_4_ nanoparticles, and pristine PU and PU nanocomposites are presented.

In the Fe_2_O_3_ and Fe_3_O_4_ nanoparticles’ spectra, the strong peaks at around 558 and 571 cm^−1^, respectively, were attributed to the vibrational frequency of the Fe–O bond [28]. In addition, the peaks observed at around 3423 cm^−1^ could be assigned to the O–H stretching vibration of H_2_O due to the moisture absorption in iron oxide nanoparticles [42,43].

The FTIR spectrum of pristine PU showed typical bands related to the polyurethane structure: at 3326 cm^−1^, the absorption peak corresponding to the N-H stretching vibration could be seen; the bands at 2940 and 2854 cm^−1^ were assigned to the asymmetric and symmetric stretching vibration of the C–H bond in CH_2_ groups; at 1731 and 1700 cm^−1^, the peaks corresponding to the free and bonded C=O stretching of urethane could be observed; the peak at 1597 cm^−1^ was due to the C=C stretching vibration of MDI; at 1532 cm^−1^, the N-H bending band was visible; the peaks at 1412 and 1310 cm^−1^ were assigned to the –CH_2_ deformation vibration; between 1107 and 1031 cm^−1^, the bands corresponding to the C–O–C stretching vibration were observable [13,31,37]. These vibration bands confirmed the formation of a pure polyurethane structure.

The addition of different iron oxide nanoparticles into the polyurethane matrix did not change the FTIR spectra of composite membranes, as can be seen in the representative IR curves [20]. This means that the molecular interaction at the surfaces was not affected by the small amounts of nanoparticles, and no physical and chemical interactions between the polyurethane chains and nanoparticles were observed. Moreover, the signals characteristic of the polyurethane structure could cover or envelop the vibration of the iron oxide structure, due to the small amount of the nanoparticles embedded in the PU matrix.

### 3.2. Mechanical Analysis

**Figure 2:**
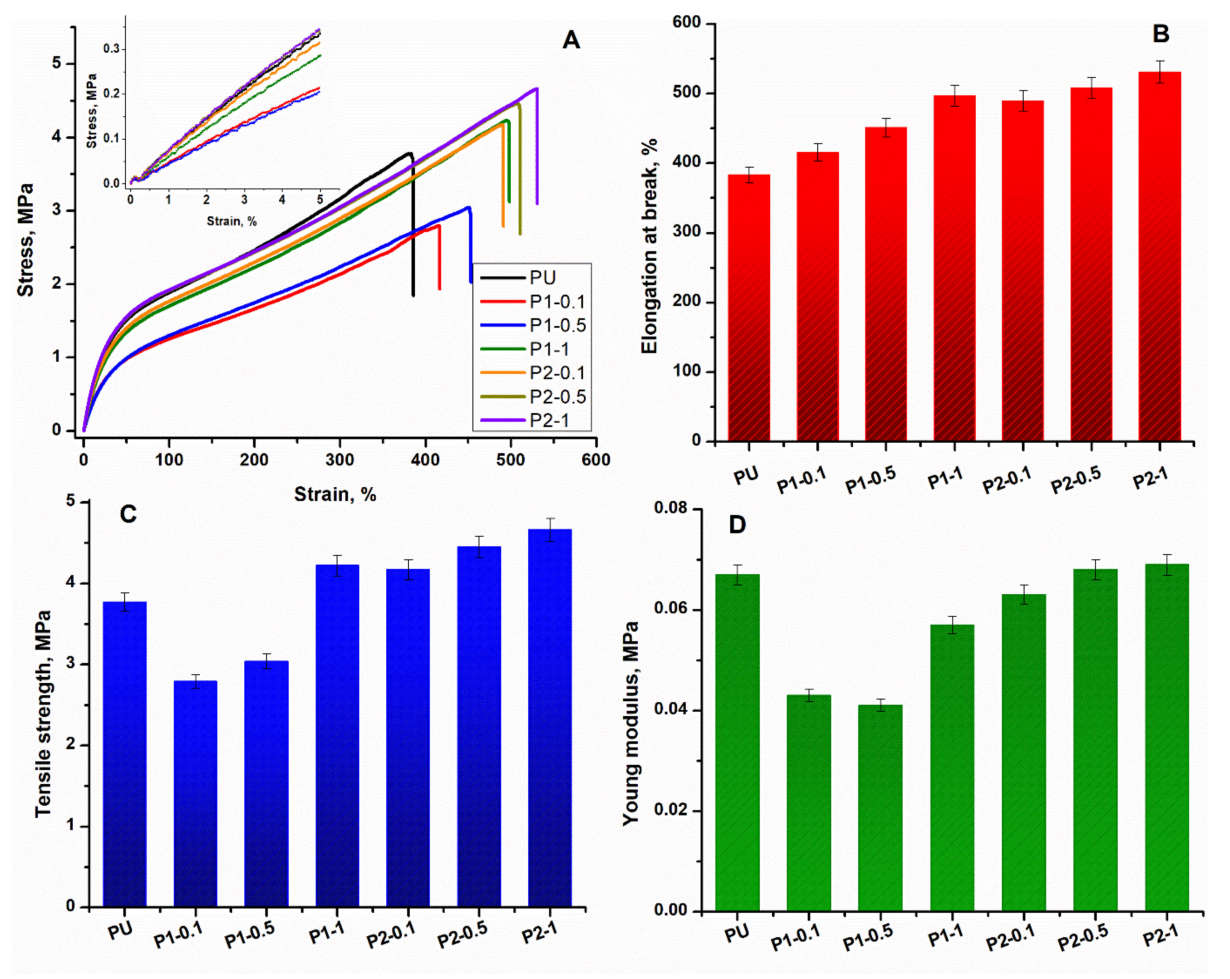
Mechanical analysis of the pristine and PU nanocomposite samples: (**A**) stress–strain curves. Inset: stress–strain curves in elastic region; (**B**) tensile strain; (**C**) tensile strength; and (**D**) Young’s modulus.

Since we aimed to explore these composite matrices as custom-tailored materials for the preparation of some suitable devices to improve the image quality in MRI investigations, mechanical properties were considered an important issue. In general, these composite materials should have an improved elasticity and strength in order to enable the high performance of devices. Thus, the mechanical properties of pristine and PU nanocomposites were also investigated by tensile tests. The effects of the incorporation of different iron oxide nanoparticles as well as the amount of nanoparticle content on mechanical properties are illustrated in Figure 2. All the curves exhibited the same pattern, showing the characteristic of an elastomeric behaviour in three stages: an elastic deformation occurred firstly, then a stress hardening took place, and finally the curves stopped when the samples broke. As can be observed in Figure 2A, the strain percent increased with the nanoparticles’ concentration and the tensile stress had a slight increase. For the samples P1-0.1 and P1-0.5 a decrease in the tensile strength was observed, meaning that these needed less stress to break, which could be attributed to different parameters such as the aggregation of the nanoparticles and the non-homogeneity of the samples [30,44]. A poorer dispersion of nanoparticles can result in more defects in the composites and, thus, can lead to poorer mechanical properties, as observed for these two samples. This behaviour was also observed in the elastic domain at low deformation of the graph (inset of the Figure 2A).

The elongation at break increased from 380% at pristine PU up to 490% for the samples embedded with 1% Fe_2_O_3_, and up to 530% for the samples with 1% Fe_3_O_4_, respectively (Figure 2B). Thus, these results show that the elongation of the samples changed as a function of the iron oxides concentration. 

Generally, the tensile strength also had a slight ascending trend with the increase in the nanoparticles’ concentration (Figure 2C). The exceptions were the samples P1-0.1 (2.79 MPa) and P1-0.5 (3.04 MPa), which had lower tensile strength values compared with pristine PU (3.77 MPa). This behaviour could be due to the aggregation of the iron oxide nanoparticles, which created different stress concentration points [20]. As a result, the interactions between the agglomerated iron oxide nanoparticles and the PU matrix were not so strong as to overcome the applied stresses during the mechanical test [45]. Moreover, the addition of these iron oxide nanoparticles led to an increase in strength, especially for nanoparticles with high surface areas (Fe_3_O_4_), as suggested by Fu et al. [46].

The Young’s modulus is the ratio between the stress and strain of a material in the elastic domain at low deformation [46]. The results showed a slight decrease for the PU nanocomposite samples (0.057 MPa at P1-1 and 0.067 at P2-1) with respect to the values of the pristine PU matrix (0.068 MPa), with the same exception for the samples P1-0.1 (0.043 MPa) and P1-0.5 (0.041 MPa), as was also previously observed for the tensile strength values (Figure 2D). This means that the samples with low modulus values could easily deform, were more elastic and presented a higher elongation until breaking. Regarding the influence of the nanoparticles’ concentration, an increase in the modulus was observed with the increasing of the nanoparticle concentration.

### 3.3. Surface Morphology of the Samples

**Figure 3:**
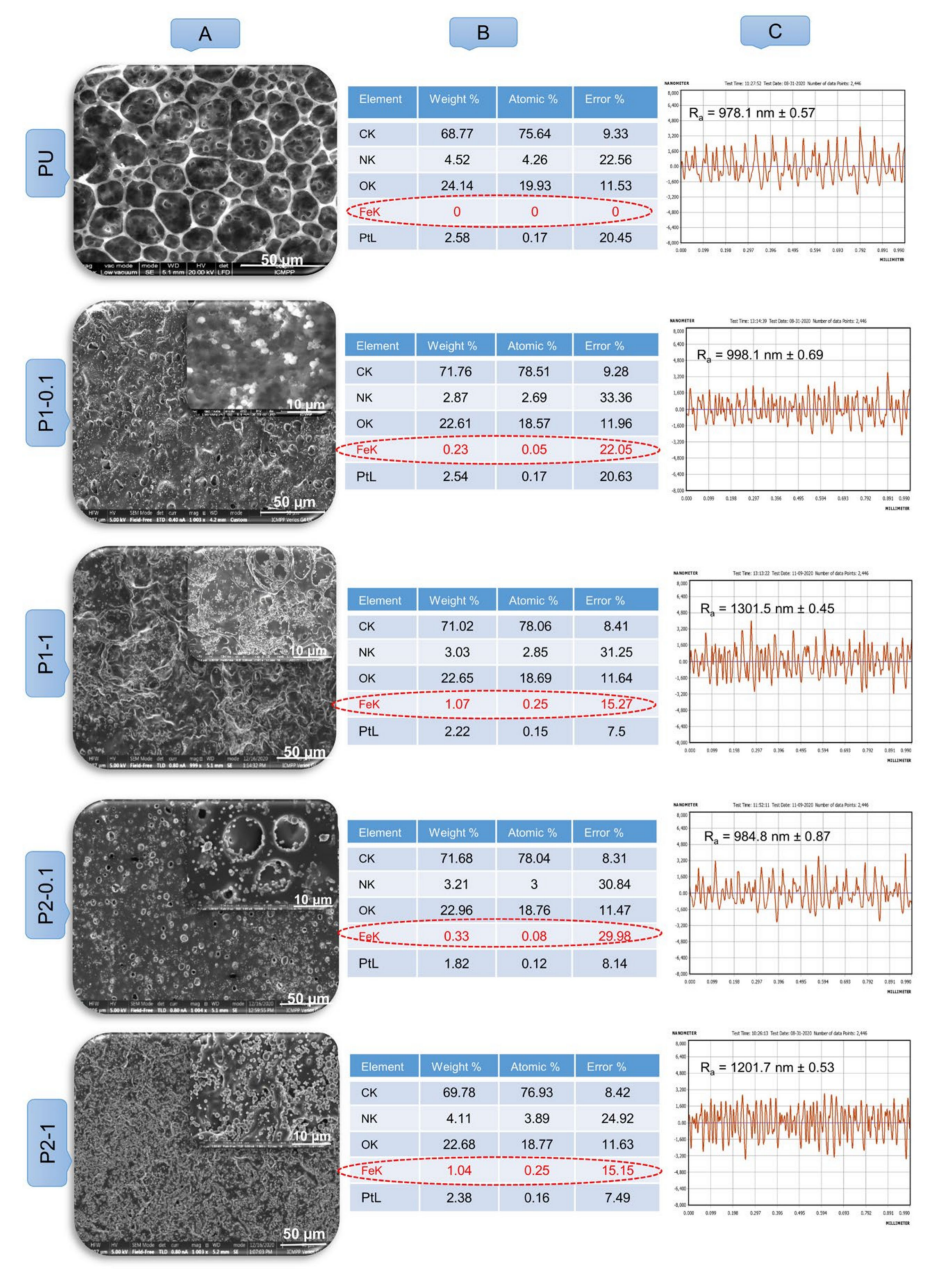
(**A**) Morphology from SEM images at 10 and 50 µm; (**B**) elemental composition; and (**C**) profilometry roughness of pristine and nanocomposite PU samples.

The surface morphology and distribution of the nanoparticles are another important factor that can affect the quality and performance of the composite materials. In order to observe the microscale surface morphology of the pristine and nanocomposite PU samples, SEM images were obtained (Figure 3A). As can be seen, the surface of the pristine PU possessed an overall uniform surface morphology with small and large pores between 5 and 20 µm, which were interconnected. When Fe_2_O_3_ and Fe_3_O_4_ nanoparticles were added, the surface morphology was changed. As shown in the SEM images, diverse surface morphologies and possibly different surface-pertaining attributes arose from different nanoparticle concentrations. Thus, for the samples with low amounts of nanoparticles (P1-0.1 and P2-0.1), some pores could be observed and the nanoparticles were quite uniformly distributed. When the concentration increased (P1-1 and P2-1), the pores were collated and the nanoparticles were sometimes agglomerated (P1-1).

To study the presence and distribution of the hematite and magnetite nanoparticles on the surfaces, the elemental composition was examined under the same operational parameters and the results are shown in Figure 3B. It was found that increasing the nanoparticle content from 0.1 to 1% resulted in an increase in the amount of them at the surfaces, for example, from 0.23 (%wt) to 1.07 (%wt) for the samples with ferric oxide nanoparticles and from 0.33 to 1.04 (%wt) for the samples with magnetite nanoparticles.

### 3.4. Profilometry Analysis

Nanoscale topography is a complementary parameter of surface characterization, which affects the functional properties of the surfaces, including friction, wettability, biological response, etc. The surfaces of the prepared samples were studied by profilometry analysis and the corresponding images are shown in Figure 3C. The average roughness (Ra) values obtained from the analyses are also given in Figure 3C for each sample. By increasing the nanoparticle content from 0 to 0.1 wt%, the Ra increased to its maximum value of 998.1 nm for sample P1-0.1 and 984.8 nm for P2-0.1, respectively, and further increased up to 1301.5 nm (P1-1) and 1201.7 nm (P2-1) for the samples with 1 wt% nanoparticles. It can also be observed that the nanoparticles’ size had a small influence on the roughness. Thus, small nanoparticle sizes (Fe_2_O_3_ of 50 nm size) led to slightly rougher surfaces than those of the samples in which the magnetite nanoparticles (Fe_3_O_4_ of 50–100 nm size) were included.

### 3.5. Wettability and Surface Energy Parameters Study

Surface wettability is a physical property that governs the interaction between the solid and liquid phases and is important in various biological systems and technological applications. Biomaterials, for example, must have hydrophobic surfaces in some applications such as those related to blood vessels [47,48], and should be relatively hydrophilic in tissue engineering [49]. It is also one of the parameters that controls cell behaviour via protein adsorption at the material surface [50].

**Figure 4:**
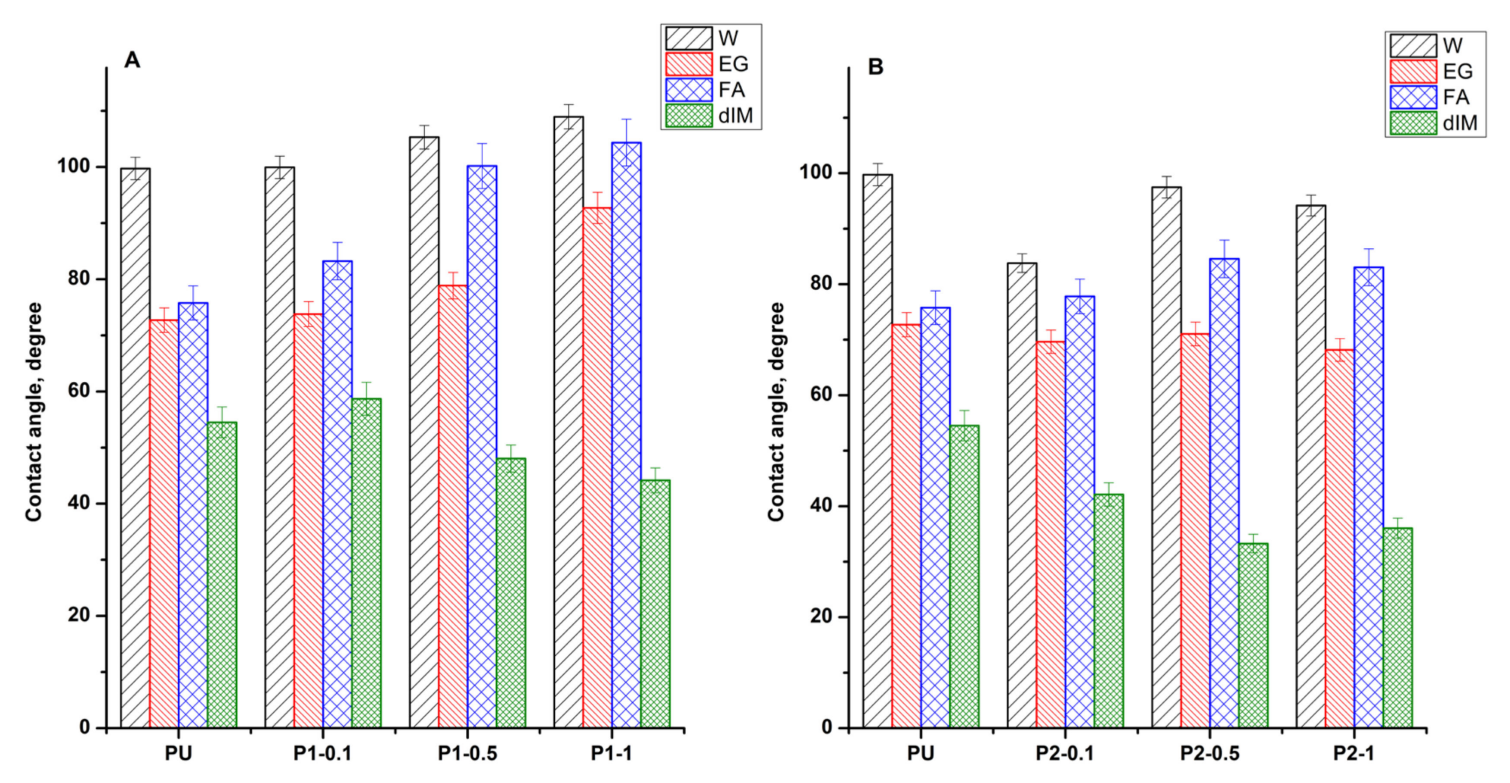
Static contact angle of pristine and PU nanocomposites: (**A**) with Fe_2_O_3_; (**B**) with Fe_3_O_4._

Investigation of the surface wettability of pristine PU and PU nanocomposites was performed by static contact angle (SCA) measurement between different polar and apolar testing liquid drops (W, EG, FA, dIM) and the surface of the samples. The contact angle values of pristine and nanocomposite PU samples, comprising different weight fractions of Fe_2_O_3_ and Fe_3_O_4_, are reported in Figure 4. Theoretically, a surface with an SCA lower than 90° is hydrophilic and, in contrast, surfaces with static SCA greater than 90° are hydrophobic. The results showed that the inorganic filler, i.e., iron oxide nanoparticles, influenced the PU surface in a different way. Thus, the incorporation of Fe_2_O_3_ nanoparticles increased the SCA of water, from 99.74° for the pristine sample (PU) up to 108.96° for the sample with 1% concentration (P1-1). This behaviour was due, on the one hand, to the increase in the roughness (Figure 3C) of the surfaces, when air pockets were trapped in the rough cavities, leading to an increase in the surface hydrophobicity [51]. On the other hand, this increase in water SCA values was due to the increase in the Fe_2_O_3_ nanoparticles’ concentration from 0.1 (P1-0.1) to 1% (P1-1) [52].

On the contrary, the presence of the Fe_3_O_4_ nanoparticles led to a decrease in the water SCA until 83.80° for the sample P2-0.1. This effect can be explained by the differences in the particles’ sizes, which is also in agreement with the study performed by Kirchberg et al. [14]. Therefore, if we compare the SCA values obtained using different nanoparticle sizes at equal concentrations (e.g., 0.1% of Fe_2_O_3_—sample P1-0.1; and of Fe_3_O_4_—sample P2-0.1), it can be observed that the incorporation of small nanoparticles (Fe_2_O_3_ of 50 nm size) led to a rough surface (Figure 3C) that corresponded to water repellence at the surface. When larger nanoparticles (Fe_3_O_4_ of 50–100 nm size) were dispersed in the PU matrix, a decrease in the water SCA was observed. 

A similar trend was observed when the PU surfaces were in contact with other polar liquids such as ethylene glycol or formamide. In contrast, because of the high-dispersive component value, methylene iodide wet the surface easily and the SCA values were smaller.

The strength of binding between the phases could be assessed by determining the work of adhesion (*W_a_*), which was dependent on the surface tension of the tested liquid and its contact angle on the surfaces (Equation (3)) [35,38]. The calculated values of the *W_a_* are illustrated in Table 2.

The values of the *W_a_* measured with a nonpolar liquid (dIM) were higher than those measured with polar liquids, due to the strong interactions between the liquid and surface. Instead, when polar test liquids were used, the PU interface decreased the solid–liquid contact area due to the less wettability, and, therefore, decreased the *W_a_* [53].

In addition, the wettability of these PU samples was also described by the spreading coefficient (*S*), a quantitative measure that represents the difference between the work of adhesion (*W_a_*) (solid–liquid interaction) and the work of cohesion (*W_c_*) (liquid–liquid interaction) (Equation (4)) [39]. *W_c_* was calculated based on this equation and was found to be 145.6, 96, 116.4, and 101.6 mN/m, respectively, for W, EG, FA, and dIM, respectively. The values of the *S* parameters are also illustrated in Table 2. This coefficient predicted the partially or totally wetting of surfaces by a liquid. As can be observed, the values were negative for all tested liquids, indicating that the liquids did not spread across the PU surfaces. This was due to the fact that the values of the *W_c_* were higher than those of *W_a_* and, thus, the liquid drops partially wet the solid surfaces. 

The surface properties of the solids and, especially, the surface free energy (SFE) with its components, are the key to understanding the mechanism of surface-based phenomena, which are crucial in different applications including adhesion, coating, printing, etc. To determine the SFE of the PU samples, along with the polar and dispersive contributions, the values of the SCA measured with the test liquids were inserted into the evaluation method formulated by Owens, Wendt, Rabel, and Kaelble [35]. The SFE values with their polar and dispersive components of pristine and nanocomposite PU samples were calculated based on the Equations (1) and (2) and are illustrated in Table 3. The results indicated a significant difference between the dispersive (*γ^d^_s_*) and the polar component (*γ^p^_s_*), the dispersive contributions exceeding the polar components at all the studied samples, confirming the hydrophobic character of the surface samples. The polar component contribution to the overall SFE was close to zero due to the dominant arrangement of nonpolar species of the PU structure on the surfaces and due to the different Van der Waals interactions between the drop liquids and surfaces (Table 3). Thus, the existence of polar and non-polar groups on the surface directly influenced the wettability property. Generally, the solids that have lower surface free energies (*γ_s_*) exhibit higher values of water contact angles [35], as was also revealed in our results. The values of *γ_s_* decreased (~26–27 mN/m at PU with Fe_2_O_3_—P1 samples) or increased (~31–34 mN/m at PU with Fe_3_O_4_—P2 samples) when compared with pristine PU (30.55 mN/m), depending on the experimental SCA values. The interfacial solid–liquid tension (*γ_sl_*) values increased with increasing contact angle values. These values can be high or low, depending on the attractive forces between the molecules of the liquid and solid [54].

Zisman [35] showed that the cosines of the SCA formed by drops of liquids on a solid surface vary linearly with their surface tensions. The critical surface tension (*γ_c_*) can then be found by extrapolating the linear function to cos *θ* = 1, and represents the maximum surface tension of a liquid that could completely wet the substrate [18]. The calculated values are given in Table 3. These values also depended on the experimental SCA values and presented the same increasing or decreasing trend as for the calculated SFE.

### 3.6. Determination of Dynamic Vapours’ Sorption and Diffusion Coefficients 

**Figure 5:**
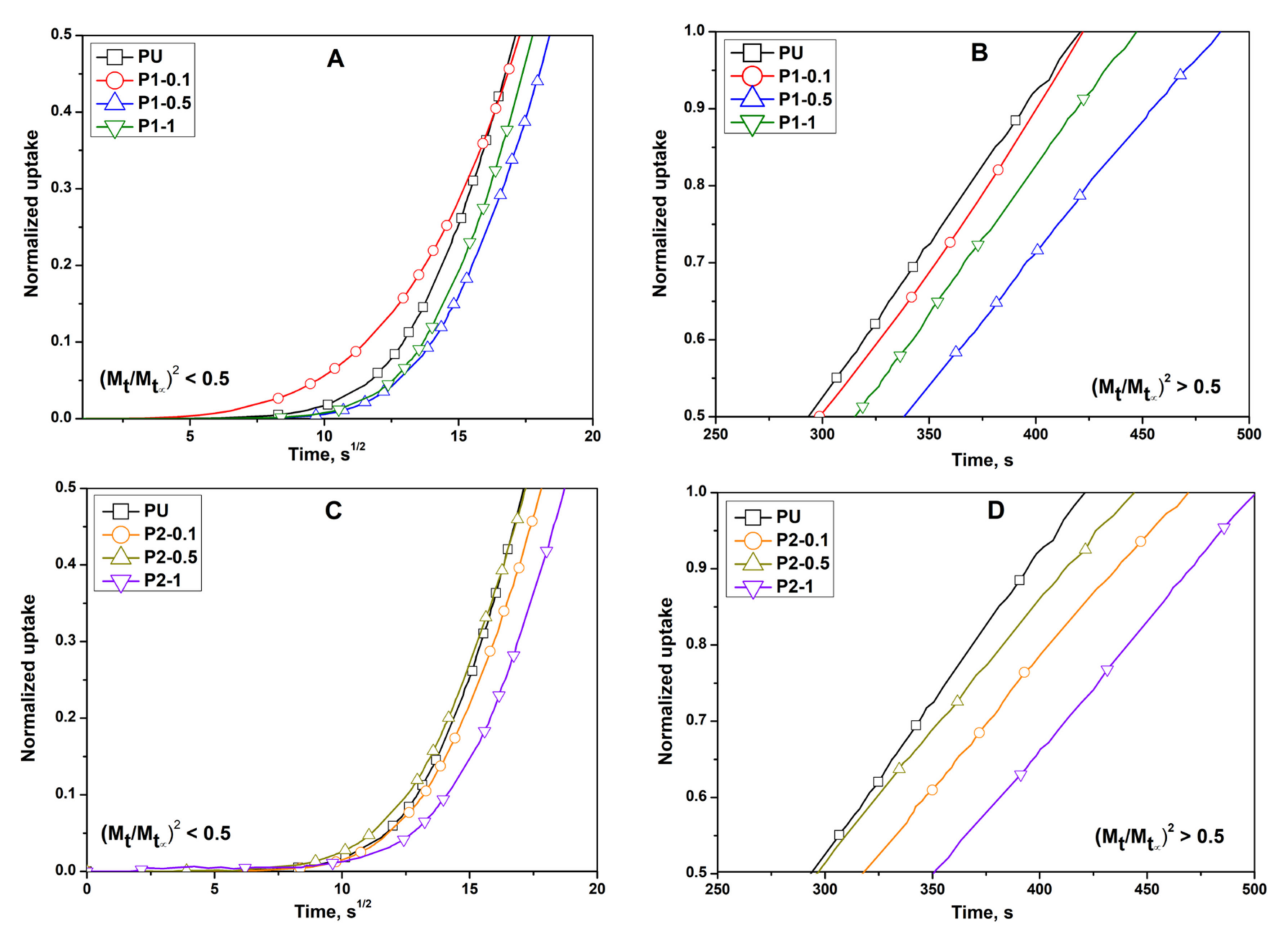
Normalized mass change as a function of time over (**A**,**C**) short time periods and (**B**,**D**) long time periods for the pristine and PU nanocomposite samples.

The sorption behaviour in polymers is a quite complex phenomena and the intensity depends upon the nature of the polymer (similar to polar/non-polar interactions) and the employed conditions. The water sorption capacity can provide important information about the prepared materials due to the fact that it is a critical factor in the determination of their stability, processing, storage, and applications. Moreover, the composite materials are exposed to water and humidity in their daily use and the attack of water and water vapour could lead to some modification of the product. This technique was used to determine the diffusion coefficients (D) of the samples using the methods developed by Crank [40] and Balik [55]. Thus, D was calculated from the plots of normalized mass change vs. time. The graphs are depicted in Figure 5, for both short and long time periods.

Therefore, over short time periods (*M_t_/M_∞_* < 0.5), the initial kinetics of sorption into the polymer could be described by equation 5 and, for long time periods (*M_t_/M_∞_* > 0.5), the kinetics could be described by equation 6. The calculated values are illustrated in Table 4. Thus, over short time periods (Figure 5A,C), the values of *D*_1_ increased with the increasing of the nanoparticle content in the composite membranes, except when the concentration of nanoparticles was 1%, in which cases these values were smaller than those of pristine PU for both P1-1 and P2-1. The increase could be partially explained by the polar nature of the iron oxide nanoparticles, which could have led to several other sites of interaction with water vapour molecules inside of the membranes, making diffusion easier. The observed deviation in the samples with high amounts of nanoparticles was possibly due to the non-homogenous distribution of the iron oxide nanoparticles in the composites (Figure 3A). Therefore, the void spaces around particles were not connected to each other in the same manner in each sample, leading to a decrease in *D*_1_.

Over long time periods (Figure 5B,D), the values of *D*_2_ were much higher than *D*_1_ mainly due to the fact that the structures had enough time to expand, leading to an increase in the diffusion process. The diffusion decreased with the increasing of the nanoparticle content in the composite membranes, with the same deviation being maintained for the samples with high amounts of nanoparticles that were observed over short time periods. This behaviour could be attributed to the possible formation of some three-dimensional coordination complexes between iron oxides and polyurethane structures, leading to some crosslinking structures [56]. These would have prevented the rearrangement of the macromolecular chains during water vapour ingression and reduced the free volume in the composite, thereby causing resistance to the penetrants, resulting in a decrease in the diffusion process.

### 3.7. Dielectric Properties

**Figure 6:**
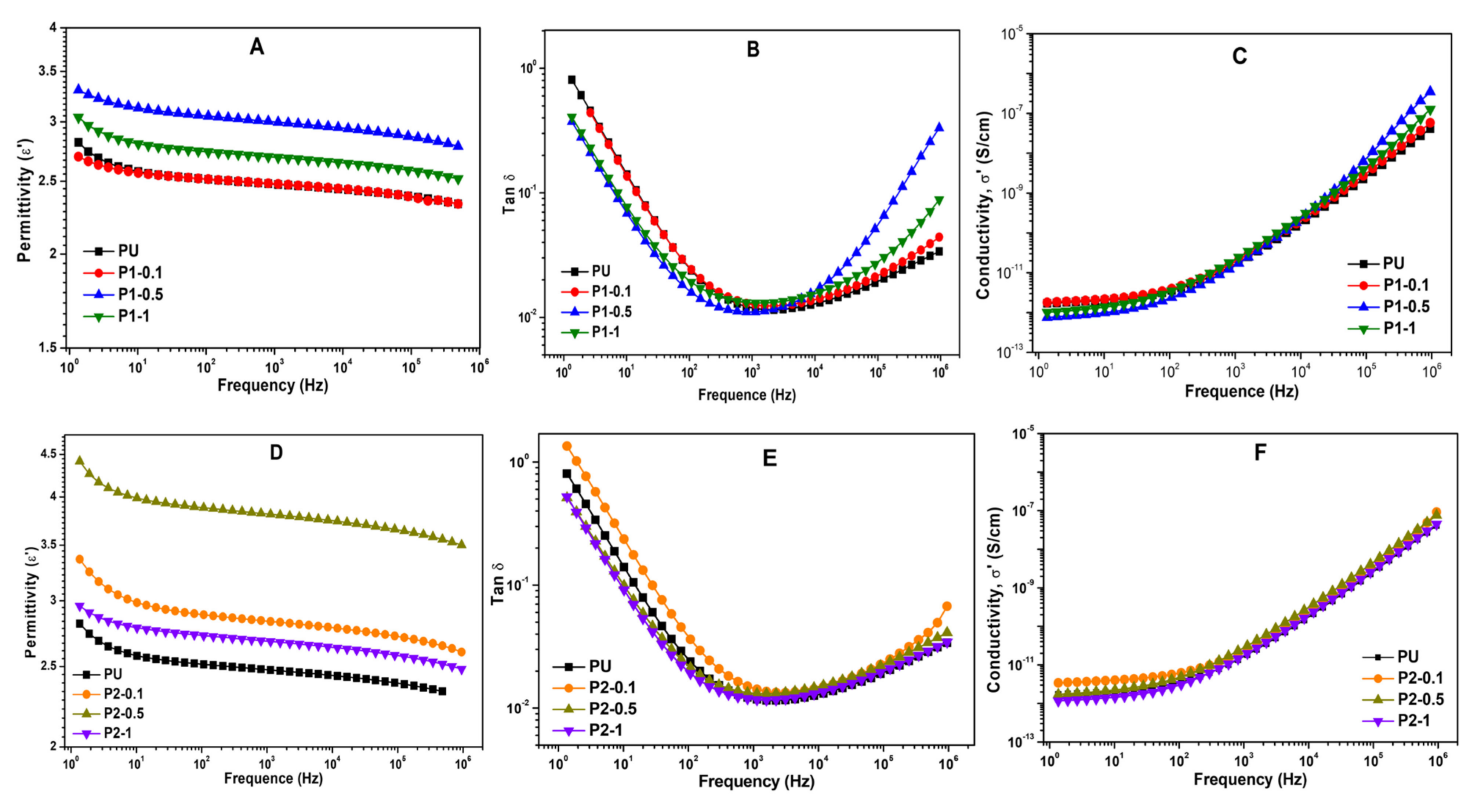
Variation of permittivity (ε’) (**A,D**), dissipation factor (tan δ) (**B,E**), and conductivity (σ’) (**C,F**) with frequency of pristine and PU nanocomposites.

The dielectric characteristics of polymer composites doped with iron oxide nanoparticles are of interest in relation to the construction of certain devices with applications related to biomedical engineering, microelectronics, nanotechnologies, etc [57,58,59,60]. Thus, the influence of different iron oxide nanoparticle contents on the dielectric behaviour of pure and PU nanocomposites was measured by dielectric spectrometry. According to the destination of the composite materials, a higher interfacial and dielectric polarisation was needed. When exposed to an electric field, the dielectric constant of the material, the so called permittivity (ε’ ‘), characterized the polarisation phenomena. The permittivity evolution, as a function of frequency, for pure and PU nanocomposites is shown in Figure 6. The permittivity values for the PU nanocomposites with Fe_2_O_3_ (Figure 6A) and for the PU nanocomposites with Fe_3_O_4_ (Figure 6D) suggested a small dependence on frequency in the range of 10^1^–10^6^ Hz. However, it should be noted that the values for P1-0.5 and P2-0.5 were very high, and quantities of iron oxide nanoparticles amounting to more or less than 0.5% tended to lower the interfacial polarisation and diminish the dielectric characteristics. 

The conductivity (σ’) of pure and PU composites (Figure 6C,F) increased slowly at low frequencies (10^0^–10^2^ Hz); however, the increase was more accentuated up to frequencies of 10^6^ Hz. It was observed that the PU composites’ conductivity curves, over the entire frequency range (10^0^–10^6^ Hz), were close to the conductivity of the pure PU curve. The presence of Fe_2_O_3_ and Fe_3_O_4_ nanoparticles introduced charge carriers into the PU matrix, and the relocation of the charge on the polymer chain helped to increase the conductivity, especially in the high frequency area, where the nano-dimension of the particles played a more important role. 

The electrical energy stored by the material was characterized by the variation of tan δ (Figure 6B,E). The values of tan δ at lower frequencies were related to an important interfacial polarisation phenomenon. The slight increase in tan δ at higher frequencies was explained by dielectric polarisation combined with an increase in conductivity. The samples with 0.5% Fe_2_O_3_ (P1-0.5) and Fe_3_O_4_ (P2-0.5) showed good behaviour in terms of the values and evolution of tan δ, but they did not reach the percolation point. 

Generally, for a material that is intended to be used for MRI applications, both a higher dielectric constant and a higher tan δ value are required. We anticipate that by processing polymer composite membranes with ferromagnetic nanoparticles that are pre-oriented in the electric field, it is possible to obtain a higher and longer-lasting remnant polarization. From a technological point of view, when a pre-orientation of the electric charged nanoparticles takes place, this membrane must be kept under a pre-defined electric field; afterwards, the structure must be “frozen” in that position by increasing the temperature and evaporation of the solvent [61]. Thus, in order to obtain excellent dielectric properties, the nanoparticles’ contents must be chosen very carefully in terms of their type, dimensions, and percentages. It is also important to achieve a pre-orientation of the nanoparticles with the use of specialized technology under a pre-defined electromagnetic field.

### 3.8. Magnetic Properties

**Figure 7:**
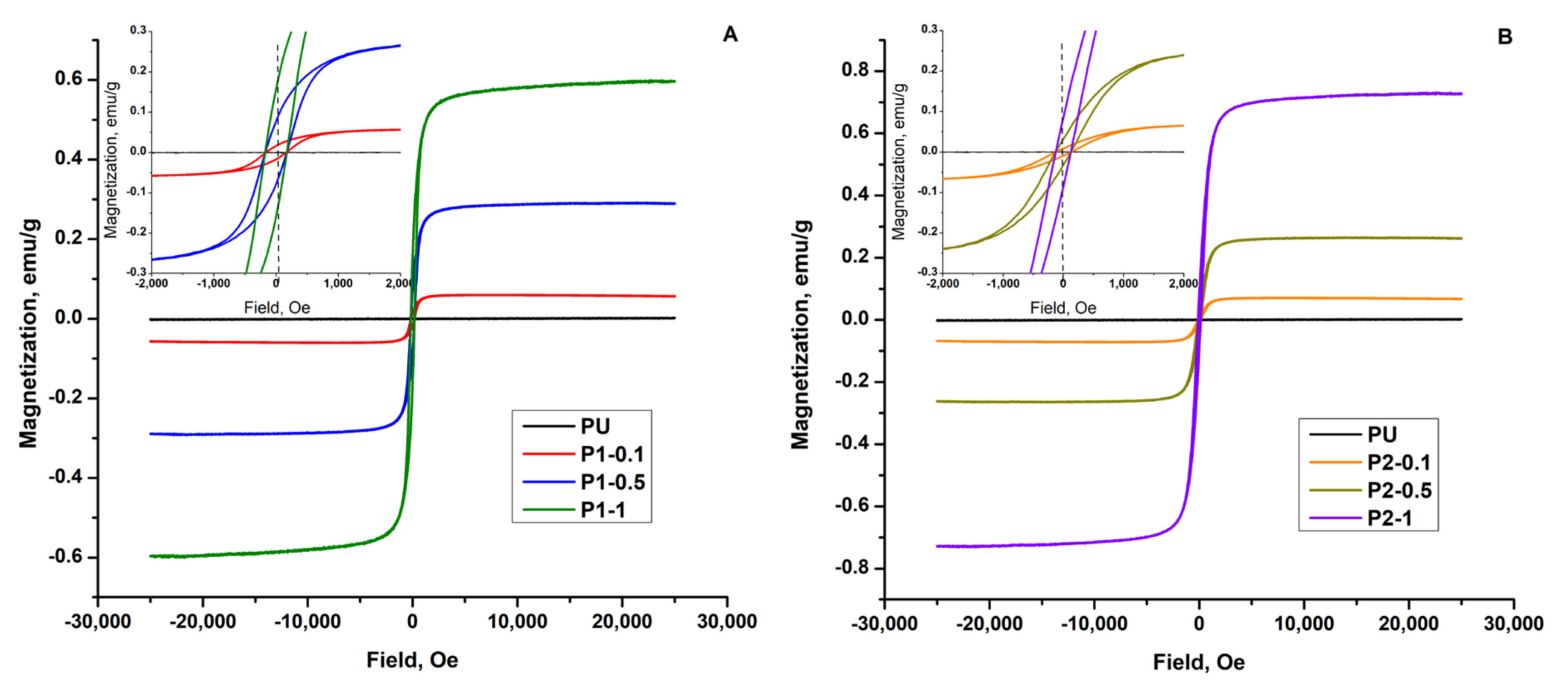
Magnetic hysteresis loops of pristine and PU nanocomposites at room temperature: (**A**) with Fe_2_O_3_; (**B**) with Fe_3_O_4_. The inset shows the hysteresis loops at low applied magnetic fields.

In addition to their aforementioned properties, these composites could be used to improve the quality of MRI devices in biomedical applications. In this regard, the magnetic properties of the pristine and nanocomposite PU samples were studied in order to evaluate the influence of the content of iron nanoparticles on the well-known magnetic behaviour of iron. Figure 7 discloses the hysteresis loops of pristine and PU nanocomposite samples registered at room temperature. The pristine PU did not show magnetic properties due to the absence of magnetic particles in the matrix. The magnetization curves of the PU nanocomposites revealed a typical S-shaped superparamagnetic behaviour with negligible magnetic coercivity and remanence, i.e., the hysteresis loops exhibited a very low remanence effect at a low applied magnetic field (inset of Figure 7) [22]. The saturation magnetization of the PU nanocomposites was quite dependent on the content of iron oxide nanoparticles. Thus, the samples with low contents of iron oxide nanoparticles (P1-0.1 and P2-0.1) displayed a low saturation magnetization of 0.056 and 0.067 emu/g, respectively. As expected, the saturation magnetization values gradually increased with the increasing of the loading of the iron nanoparticles, until values of 0.583 and 0.728 emu/g were reached for P1-1 and P2-1, respectively. However, the smaller saturation magnetization values of nanocomposites, as compared with those of the pure nanoparticles, were due to the low iron oxide content in the PU matrix (max 1 wt.%), the smaller nanoparticle sizes (<50–100 nm), and also to the surface coating of the iron oxide nanoparticles by the polymer chains [44,62,63]. The saturation magnetization was reached at a field of approximately 2000 Oe [64].

### 3.9. Cytotoxicity Screenings and In Vitro Evaluation of Biocompatibility

**Figure 8:**
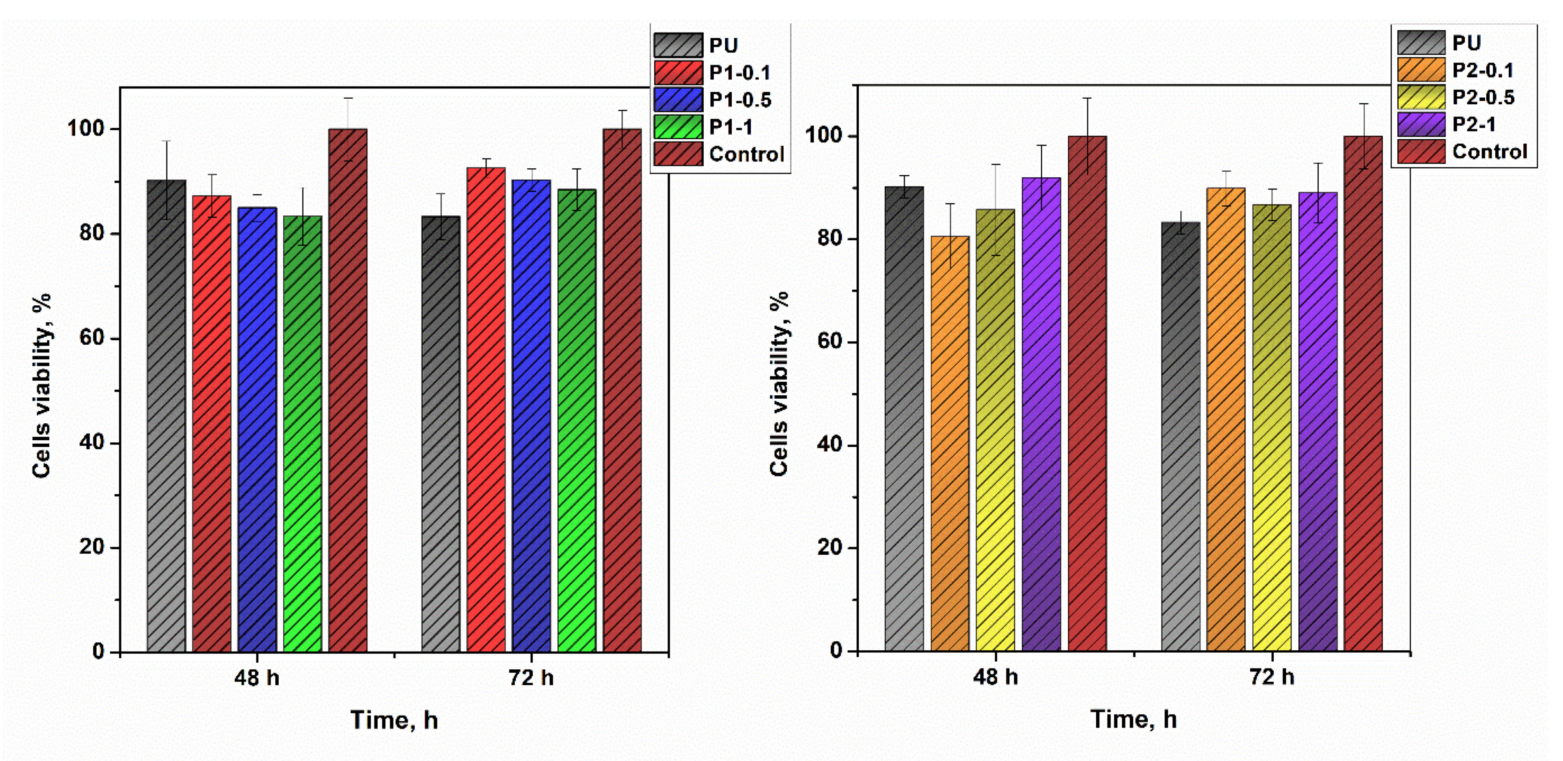
MTT assay showing the viability of epithelial cells in the presence of the pristine and PU nanocomposite samples at 48 and 72 h.

The cytotoxic effects of pristine and PU nanocomposite samples were evaluated by measuring the metabolic activity of epithelial cells using an MTT assay. Generally, cytotoxicity can be established on the basis of cell viability comparative to controls. Thus, activity levels of less than 30% are considered as representing severe cytotoxicity, values of between 30 and 60% denote moderate cytotoxicity, activity of between 60 and 90% indicates slight cytotoxicity, and for values greater than 90%, it is assumed that a material is not cytotoxic [65,66]. Figure 8 shows the results of MTT assay for pristine and PU nanocomposite samples after 48 and 72 days of contact with epithelial cells. Compared to the control, the PU nanocomposite samples (P1-0.1, P1-0.5, P1-1, P2-0.1, P2-0.5, P2-1) maintained their cell viability at values in the range of 80–90% and it always remained higher than the viability recorded for pristine PU after 72 h. There was a decrease in the cell viability (83%) after 72 h of cell culture for the pristine samples (PU) compared to control, which can be considered as indicating very slight to no cytotoxicity.

**Figure 9:**
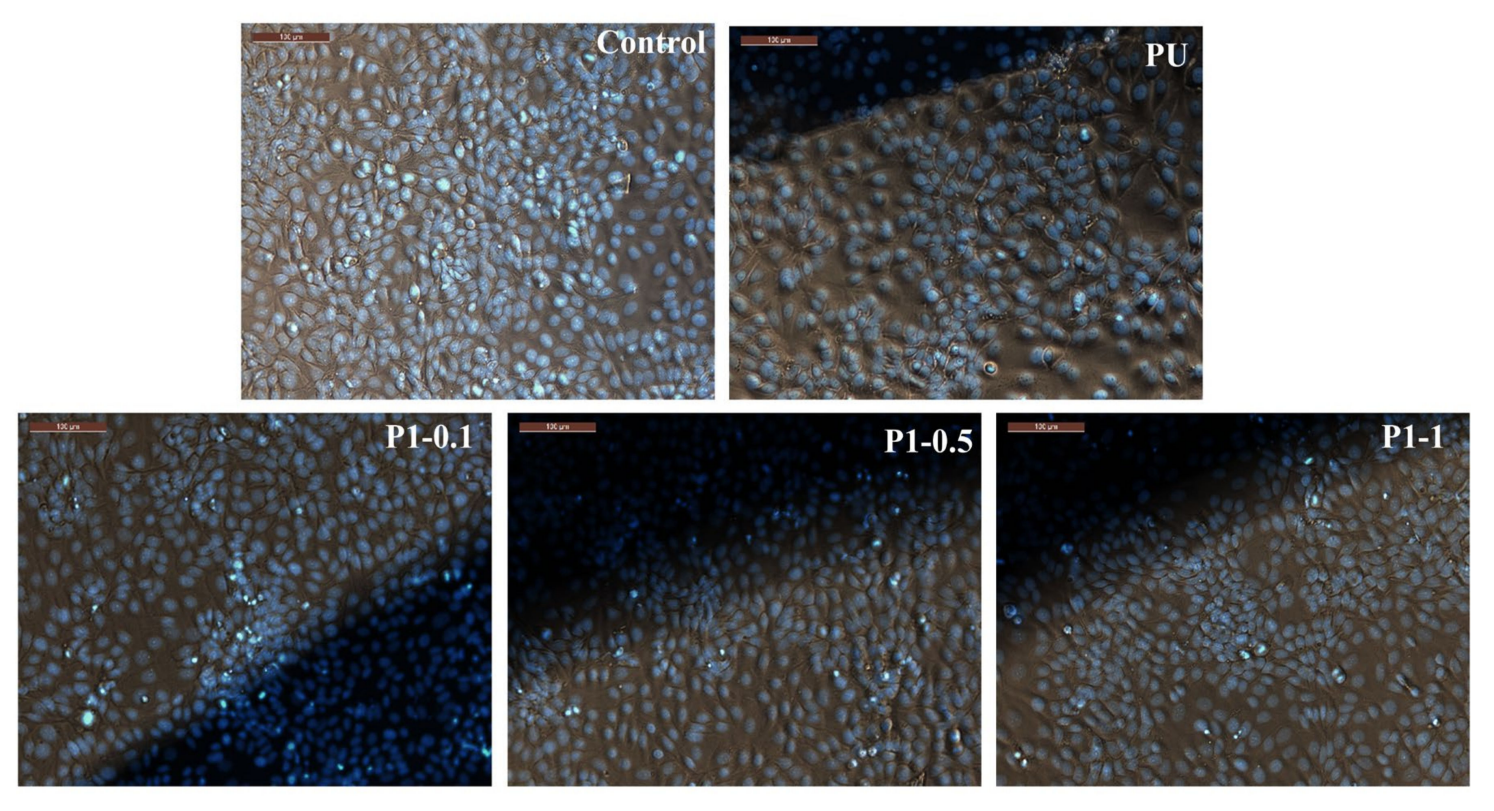
Morphological evaluation of epithelial cells after 72 h direct contact with the pristine and PU composites with different amounts of Fe_2_O_3_.

**Figure 10:**
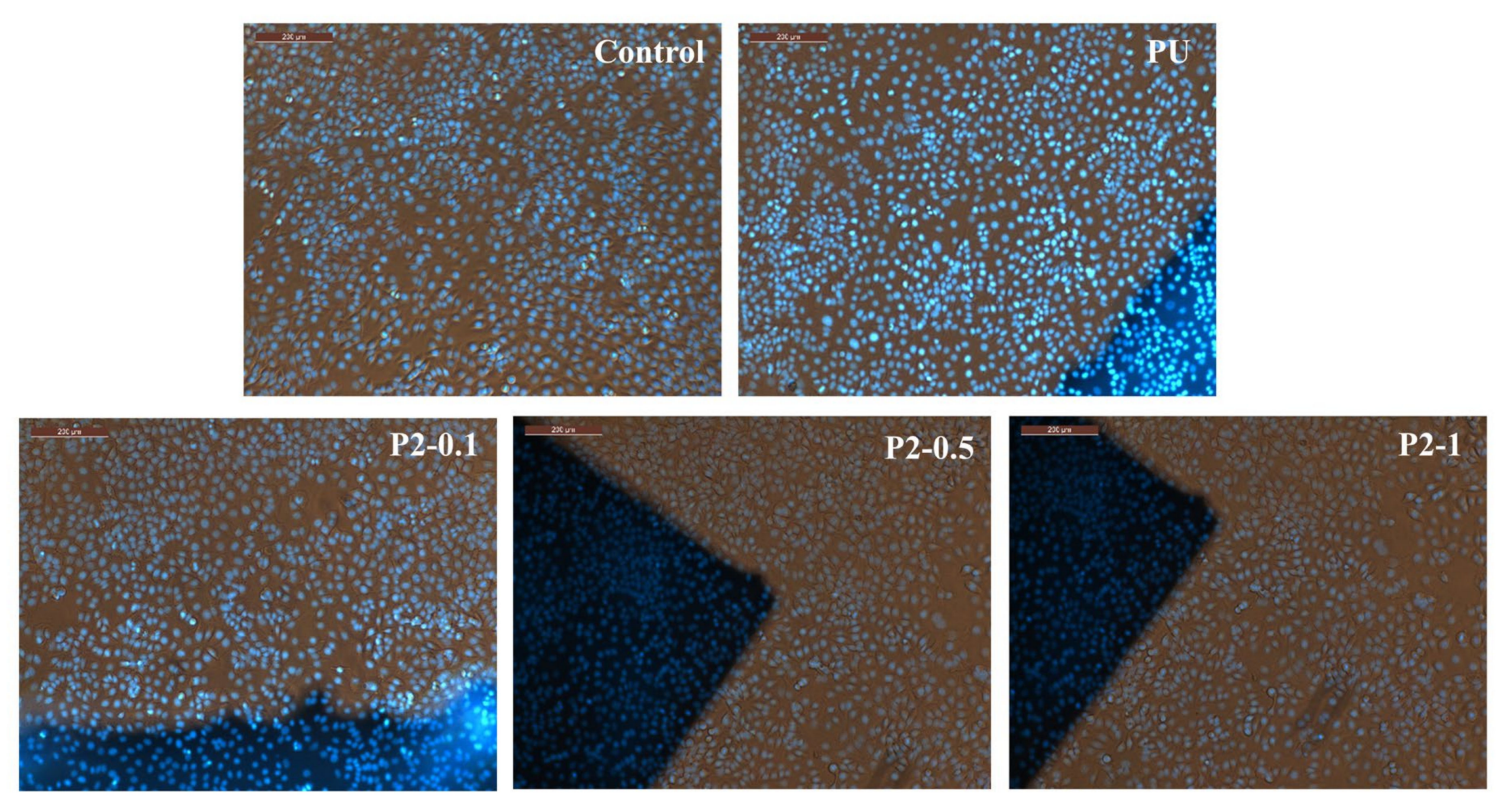
Morphological evaluation of epithelial cells after 72 h direct contact with the pristine and PU composites with different amounts of Fe_3_O_4_.

The biocompatibility of pristine and PU nanocomposites was also investigated by means of a morphological evaluation of the epithelial cells after direct contact with the samples (Figure 9 and Figure 10). Thus, when compared with the control, no differences were observed on the cell morphology. All the analysed nanocomposite films contained epithelial cells with a typical MCF 7-cell-line shape. After 72 h of culture, the cells grew in monolayers, with a very small difference in the density between the growth control and the analysed films. The cells were evenly distributed in the culture, including the areas containing the films that appeared as darker fields in the images. P1-0.1 and P2-0.1 had the best biocompatibility properties, maintaining cell viability at almost 93 and 90%, respectively, after 72 h of cell incubation (Figure 8). Thus, the lower the iron concentration in PU nanocomposite samples, the better the non-toxicity properties. Similar behaviour was confirmed by other authors in a previous study [20], where the optimal concentration of Fe_2_O_3_ nanoparticles was found to be 0.1%. It is known that the addition of iron nanoparticles into the polyurethane matrix improves the connectivity of the cells through their receptors because the iron nanoparticles have good conductivity properties [20]. Therefore, the obtained results indicated that the PU nanocomposite samples had good cytocompatibility and could be used in the chosen biomedical applications.

## 4. Conclusions

In the present research work, magnetic nanocomposites with poly(ether-urethane) used as their matrices and two types of iron oxide nanoparticle (Fe_2_O_3_ and Fe_3_O_4_) as their nano-sized fillers were prepared and characterized. The nanocomposite PU membranes were prepared via solution casting by embedding different weight percentages (0.1; 0.5; 1%) of both Fe_2_O_3_ and Fe_3_O_4_ nanoparticles into PU matrices (30 wt.% in DMF) through blending. The effects of the amounts of nanoparticles on the various properties of the prepared PU nanocomposite membranes were investigated and compared with the results obtained for pristine PU. Thus, the chemical composition and molecular structure of the pure and PU nanocomposites were examined using FTIR-ATR analysis. The molecular interaction at the surfaces was not affected by the small amounts of nanoparticles and no physical or chemical interactions between the polyurethane chains and nanoparticles were observed. This was mainly due to the fact that the polyurethane structure could cover or envelop the vibration of the iron oxide structure. Mechanical analysis showed that the PU nanocomposites presented an elastomeric behaviour, with the elongation at break increasing from 380% for pure PU up to 490% for P1-1, and reaching 530% for P2-1. The Young’s modulus showed a slight decrease for the PU nanocomposites (0.057 MPa for P1-1 and 0.067 for P2-1) with respect to the values of the pure PU matrix (0.068 MPa), The introduction of different iron oxide nanoparticles afforded better mechanical properties of the PU nanocomposites as compared to those of the pristine PU. Different surface morphologies were observed for the pure and nanocomposite PU membranes with interconnected pores (average between 5 and 20 µm) for pure PU, and a fairly uniform distribution of nanoparticles was observed for the samples with low amounts of iron oxide nanoparticles and collated pores, and the agglomeration of nanoparticles was sometimes observed at high nanoparticle contents. The wettability of the surfaces depended on the roughness and size of the iron oxide nanoparticles. Thus, the incorporation of Fe_2_O_3_ nanoparticles increased the SCA of water, from 99.74° at pure PU up to 108.96° for P1-1. On the contrary, the presence of Fe_3_O_4_ nanoparticles led to a decrease in the water SCA to 83.80° for the P2-0.1 sample. Water vapour diffusion increased over short time periods because of the polar nature of the iron oxide nanoparticles, and it decreased over long time periods due to the formation of 3D-crosslinking coordination complexes between the iron oxides and polyurethane structures. As expected, the magnetic properties were significantly enhanced with higher amounts of iron oxide nanoparticle loading (0.583 and 0.728 emu/g for P1-1 and P2-1, respectively), when compared with pure PU. In addition, the investigation of the in vitro biocompatibility of the prepared PU nanocomposites showed that, with cell viability levels in the range of 80–90%, these samples are good candidates for biomedical applications. Considering all the investigations, we can conclude that these PU nanocomposites could be used as matrices for the preparation of certain suitable devices, particularly to improve the quality of MRI investigations.

## Figures and Tables

**Table 1:** Sample code designations and formulations of pure PU and nanocomposites.

Sample Code	Molar Ratio * (PTHF:MDI:BG/PDMS)	Nanoparticle Type	Nanoparticle Content (wt%)
PU	1:3:2	None	0
P1-0.1	1:3:2	Fe_2_O_3_	0.1
P1-0.5	1:3:2	Fe_2_O_3_	0.5
P1-1	1:3:2	Fe_2_O_3_	1
P2-0.1	1:3:2	Fe_3_O_4_	0.1
P2-0.5	1:3:2	Fe_3_O_4_	0.5
P2-1	1:3:2	Fe_3_O_4_	1

* Molar ratio of polyol:diisocyanate:chain extender.

**Table 2:** Work of adhesion and spreading coefficient values of pristine and PU nanocomposite samples.

Sample Code	*W_a_* (mN/m)				*S* (mN/m)			
	W	EG	FA	dIM	W	EG	FA	dIM
PU	60.47	62.26	72.49	80.29	−85.12	−33.73	−43.90	−21.30
P1-0.1	60.22	61.38	65.06	77.18	−85.37	−34.61	−51.33	−24.41
P1-0.5	53.57	57.27	47.90	84.76	−92.02	−38.72	−68.49	−16.83
P1-1	49.14	45.72	43.77	87.23	−96.45	−50.27	−72.62	−14.36
P2-0.1	80.65	64.68	70.48	88.47	−64.94	−31.31	−45.91	−13.12
P2-0.5	63.33	63.58	63.69	93.26	−82.26	−32.41	−52.70	−8.33
P2-1	67.47	65.84	65.22	91.87	−78.12	−30.15	−51.17	−9.72

**Table 3:** Surface tension parameters of pristine and PU nanocomposite samples calculated using the OWRK and Zisman methods.

Sample Code	*γ^d^_s_* (mN/m)	*γ^p^_s_* (mN/m)	*γ_s_* (mN/m)	*γ_sl_* (mN/m)	*γ_c_* (mN/m)
PU	30.25	0.30	30.55	38.24	26.43
P1-0.1	26.58	0.49	27.08	37.24	22.58
P1-0.5	27.50	0.02	27.52	43.03	25.61
P1-1	26.66	0.25	26.91	35.27	19.22
P2-0.1	29.09	2.60	31.69	14.78	19.85
P2-0.5	35.06	0.04	35.11	35.13	32.15
P2-1	33.87	0.32	34.19	31.35	31.49

**Table 4:** Diffusion coefficients determined from the experimental data of pristine and PU nanocomposite samples.

Sample Code	*D*_1_ × 10^−7^ (cm^2^/s)	*D*_2_ × 10^−7^ (cm^2^/s)	*K*_1_ × 10^−3^ (s^−1^)	*K*_2_ × 10^−3^ (s^−1^)	*l* (cm)
PU	0.90	7.14	1.15	−17.62	0.02
P1-0.1	1.04	5.05	1.48	−12.46	0.02
P1-0.5	1.16	5.81	1.33	−14.33	0.02
P1-1	0.76	6.42	0.97	−15.84	0.02
P2-0.1	1.05	6.22	1.34	−15.33	0.02
P2-0.5	1.13	5.59	1.44	−13.78	0.02
P2-1	0.77	5.87	0.98	−14.49	0.02

*D*_1_ is the diffusion coefficient calculated for short time periods (*M_t_/M_∞_* < 0.5); *D*_2_ is the diffusion coefficient for long time periods (*M_t_/M_∞_* > 0.5); *K*_1_ is the slope of linear regression between (t-t_R_) and (*M_t_/M_∞_*)^2^ for (t-t_R_) ≥ 0 and (*M_t_/M_∞_*)^2^ < 0.2 where t_R_ is the time correlation for *M_t_/M_∞_* = 0; *K*_2_ is slope of linear regression between t and ln(1− *M_t_/M_∞_*) for −1.2 > ln > −3.0.

## Data Availability

The data presented in this study are available on request from the corresponding author.
